# The Optimal Pain Management Approach for a Laboring Patient: A Review of Current Literature

**DOI:** 10.7759/cureus.1240

**Published:** 2017-05-10

**Authors:** Albert Kelly, Quang Tran

**Affiliations:** 1 Anesthesiology, Riverside University Health System Medical Center, Moreno Valley, California, United States

**Keywords:** labor epidural, labor analgesia, regional anesthesia

## Abstract

There is a general agreement that a patient in labor should be given the option to have an epidural block for pain management. Despite this consensus, there are differences in practice patterns as to when to initiate an epidural and how to minimize its impact on the duration and outcome of a patient’s labor. A review of the literature suggests epidural analgesia does prolong stages one and two of labor, but not significantly. Cesarean delivery rates are not affected by the early initiation of epidural analgesia. The use of various adjuvants such as opioids, clonidine, and neostigmine in conjunction with local anesthetics solution can significantly reduce the severity of motor blockade and the need for assisted vaginal delivery.

## Introduction and background

The purpose of this review is to determine a literature-guided approach for providing a safe and pleasant experience for patients. The patient’s experience is an increasing part of medical care; it is, therefore, important to adjust and shape our practice to this growing aspect of medicine. Optimal pain management for a laboring mother revolves around three main questions: (1) What is the consensus for the effects of neuraxial anesthesia on the rate of cesarean deliveries and course of labor? (2) When is the appropriate time to initiate epidural analgesia for a laboring patient? (3) What are the optimal drug combinations and concentrations that provide the best maternal and fetal outcomes?

## Review

In determining the optimal pain management regime for a woman in labor, the first question involves the impact of epidural anesthesia (EA) on the duration of labor. A retrospective case study by Hasegawa, et al. looked at the length of labor and found the “mean lengths of the 1st stage and 2nd stage of labor were 176 and 31 min in controls versus 269 and 39 min in cases” where EA was used [1]. A regression analysis revealed this prolongation was due to EA after adjusting for multiparity. Another study by Gizzo, et al. found a statistically significant difference in total length of labor (363.58 ± 62.20 minutes versus 292.30 ± 64.75 minutes in the non-EA group) [2]. In this study, they found that the mean difference in the second stage of labor is about eight minutes, which was not statistically significant. In contrast to these assertions, an experimental study out of Pakistan by Anwar, et al. found a statistically significant difference in the length of the second stage of labor (defined as a duration greater than one hour for multigravid and greater than two hours for primigravid patients) [3]. This study had 100 patients, which may make it relatively underpowered. Looking at the available data, we can surmise that EA does increase the total length of labor.

The question remains whether this increased duration of labor has an impact on the outcome. Several observational studies have suggested that there are positive effects from controlling maternal pain. In addition, there is the potential risk that a painful labor may negatively “alter maternal physiology and biochemistry” (e.g., hypertension, increases in circulating catecholamine levels, and uterine vasodilation) [1-2]. Therefore, the American College of Obstetricians and Gynecologists and the American Society of Anesthesiologists both support the premise that a “maternal request is a sufficient medical indication for pain relief during labor,” barring any contraindications [2,4]. Several observational studies suggest the rate of cesarean deliveries and assisted vaginal deliveries are increased using EA. In a nonblinded study from Saudi Arabia in 2012, Mousa, et al. compared two groups of parturients receiving either EA during labor or no analgesia at all. They enrolled patients into either group based on maternal interest in EA or not. They found “no statistical difference in the duration of the active first and the second stages of labor, instrumental delivery, vacuum-assisted or cesarean delivery rates, the number of newborns with 1-min and 5-min Apgar scores less than 7” [5]. This stance supported a Cochrane review published in 2011 [2].

In evaluating an EA’s impact on labor, another potential variable is the degree of concentration of local anesthetic used. In a meta-analysis, Sultan, et al. compared the effects of low-concentration versus high-concentration local anesthetics (LA) used in EA. The results of that analysis concluded that low concentrations of LA used in EA significantly reduced the occurrence of assisted (i.e., forceps or vacuum) vaginal delivery (AVD) when compared to high concentrations of local anesthetic (defined as >0.1% bupivacaine or >0.17% ropivacaine) [6]. This result was hypothesized due to increased motor blockade secondary to high concentrations of LA. However, multiple analyses showed no difference in cesarean delivery rates between high or low concentrations of LA [6-7]. Ismail reviewed multiple randomized clinical trials (RCTs) and concluded the results “did not demonstrate any difference in the rate of [cesarean delivery] between women receiving epidural and those who received only intravenous analgesia” [7]. With the majority of data supporting a nonsignificant correlation between cesarean delivery rates and epidural analgesia, we can confidently educate our patients and colleagues that EA should be a viable option for the majority of our laboring population.

The application of the discussion to this point raises the question of optimal timing to commence EA. Ismail referenced studies by Wong, et al. in 2005 and 2009 along with two other systematic reviews (the latest of which was in 2011) that conclude there is no effect on the rate of cesarean deliveries with early institution of EA (defined as 3 cm or less in the latent phase of labor) compared to EA started between 3 cm to 5 cm or greater than 5 cm of cervical dilation [7]. Early initiation of EA results in more potential exposure of the mother to LA, which does not seem to significantly increase the risk of a negative outcome for the mother or fetus. The results from the RCTs and meta-analyses suggest that severe pain necessitating early requests for EA may be due to inherent dysfunctional labor involving intrinsic maternal or fetal causes that lead to higher incidences of cesarean deliveries [7]. Large fetal size, small pelvic outlets, and a variety of maternal health problems may have more of a causal relationship to cesarean delivery rates than the early initiation of EA itself.

The literature suggests [1-5] that there may be an increased incidence of a prolonged second stage of labor and instrumented vaginal delivery. This leads to the question of techniques that can minimize this impact on patients.

A few studies compared various combinations of LA and opioid as well as the varying concentrations of LA that can be used for epidural analgesia. In a prospective, double-blinded RCT at Parkland Hospital in Dallas, Craig, et al. investigated a potential correlation between LA and increased motor blockade which may lead to AVD or prolongation of the second stage of labor. In a well-designed study, they randomized patients with EA already in place once they reached 8 to 10 cm in cervical dilation to either receive a solution with bupivacaine 0.125% and 2 mcg/mL fentanyl or a solution of 10 mcg/mL fentanyl alone. They concluded that neither solution significantly affected the duration of the second stage of labor (75 minutes for dual therapy, 73 minutes for fentanyl alone), degree of motor blockade, rates of spontaneous vaginal delivery, forceps-assisted delivery, or cesarean delivery rates [8]. The fentanyl-only patients received a five-fold increase in exposure to opioids; the study did not investigate side effects further.

Neostigmine and clonidine have also been under investigation as promising adjuvants of EA that can potentially increase the quality of analgesia while also reducing LA requirements. In a 2014 double-blinded RCT in Belgium, Boogmans, et al. sought to evaluate whether these adjuvant medications can improve patient satisfaction (measured by a reduction in the visual analog scale) and reduce the incidence of breakthrough pain requiring patient-controlled epidural analgesia (PCEA) with a ropivacaine/sufentanil solution [9]. The mechanism behind this combination therapy is not completely clear, but it is known that clonidine is an alpha-2-receptor agonist that can modulate pain perception at the spinal level and neostigmine (an acetylcholinesterase inhibitor) can indirectly stimulate both muscarinic and nicotinic receptors in the spinal cord [10]. Boogmans, et al. employed a combined spinal-epidural (CSE) technique where all participants received a spinal injection of ropivacaine and sufentanil initially. Patients were then randomized into a neostigmine-clonidine (NC) group receiving a 500-mcg neostigmine and 75-mcg clonidine solution. A placebo (control) group received saline. PCEA with ropivacaine and sufentanil was made available to both groups. The NC group had a significant reduction in ropivacaine use (by 32.6%), breakthrough pain (3% vs. 36%), and patient satisfaction after one hour [9]. In 2011, Loubert, et al. commented in a review article that low doses of LA can reduce the rate of AVD and provide adequate analgesia when adjuvant medications such as opioids are added. However, they noted that fentanyl and sufentanil are lipophilic, and there are some degrees of systemic absorption along with some unwanted side effects such as sedation, nausea, vomiting, and, most annoyingly, pruritus. Although they stopped short of recommending these adjuvant medications for every patient, the results were favorable, and evidence suggests that these adjuvant medications should be considered as additional options to epidural opioids [10].

No matter what the medications are used, hypotension and subsequent fetal arrhythmias are known risks at the onset of EA. To mitigate these risks, patients are usually preloaded with crystalloid solutions (500 to 1000 mL of either normal saline or lactated Ringer’s solution). However, findings from two separate prospective randomized trials in the United Kingdom by Hawthorne, et al. and Kubli, et al. contradict the previously accepted notion that preloading is required to prevent hypotension after EA placement. In each study, no statistically significant increases in rates of hypotension (>20% decrease from baseline mean arterial pressure) or fetal arrhythmias [11-12] were noted. The investigators reported they used a low concentration (0.1% bupivacaine with fentanyl) delivered via bolus while the patient was in a sitting position to ensure no aortocaval compression. This evidence would suggest that preloading may not be necessary for all patients and can be an area where the speed of EA delivery can be improved.

A concise oral presentation by Dr. Carvalho [13] during the 2016 Society of Obstetric Anesthesia and Perinatology Sol Shnider, M.D. Obstetric Anesthesia Meeting in San Francisco also helped summarized the latest technology aimed at delivering the best possible labor analgesia in the safest manner. Carvalho noted that a lower concentration of local anesthetic delivered in higher volumes achieved better uniform spread and subsequent analgesia compared to a higher concentration LA delivered at lower volumes. The benefit of the prior mode of delivery is lower LA consumption and less motor sensory block. Secondly, he stated that a technique which employs a PCEA and periodic intermittent epidural bolus provided better patient satisfaction and analgesia compared to either a continuous epidural infusion (CEI) or CEI with PCEA. He surmises that this is once again due to increasing the volume infused over a shorter amount of time. Lastly, he advocated for employing CSE techniques to expedite pain relief. Concerns over hypotension and fetal bradycardia can be mitigated by using a smaller amount of initial LA in the spinal bolus.

## Conclusions

The literature suggests four key points that can be employed to deliver a safe, timely, and effective labor analgesia to our patient population. First, an epidural should be considered and provided whenever the laboring patient requests it, as long as no contraindication is found. Early initiation does not increase maternal or fetal outcomes and delaying anesthesia consultation for these patients should be considered a disservice. Next, a solution with a lower concentration of LA with either opioids or possibly neostigmine/clonidine as adjuvants may provide superior analgesia compared to a solution of LA alone. By doing this, there will be less motor blockade which may interfere with the expulsion forces needed during the second stage of labor and hopefully decrease the rate of AVD. Thirdly, it may not be necessary to delay epidural placement while waiting for a crystalloid preloading to be completed. Lastly, and most importantly, PCEA will result in better patient satisfaction while also decreasing unnecessary amounts of medications needed to achieve an acceptable level of analgesia.
